
JOURNAL INFORMATION
==============================
NLM Title Abbreviation: Biospektrum (Heidelb)
Journal ID: Biospektrum (Heidelb)
NLM Journal ID: 9615685

Biospektrum

ISSN: 0947-0867
EISSN: 1868-6249

ARTICLE INFORMATION
==============================
PMCID: PMC8233622
PMID: 34219981
DOI: 10.1007/s12268-021-1595-3
Article ID: 1595
Article version: 1
Subjects: Wissenschaft

SARS-CoV-2 Nsp1: Ein kleines Protein blockiert die Immunantwort

Sparrer Konstantin Konstantin.Sparrer@uni-ulm.de

Kirchhoff Frank Frank.Kirchhoff@uni-ulm.de

grid.410712.1 Institut für Molekulare Virologie, Universitätsklinikum Ulm, Meyerhofstraße 1, D-89081 Ulm, Deutschland
Electronic publication date: 2021 Jun 26
Print publication date: 2021
Volume: 27
Issue: 4
First page: 368
Last page: 371
Copyright: © Die Autoren 2021
License: Open Access: Dieser Artikel wird unter der Creative Commons Namensnennung 4.0 International Lizenz veröffentlicht, welche die Nutzung, Vervielfältigung, Bearbeitung, Verbreitung und Wiedergabe in jeglichem Medium und Format erlaubt, sofern Sie den/die ursprünglichen Autor(en) und die Quelle ordnungsgemäß nennen, einen Link zur Creative Commons Lizenz beifügen und angeben, ob Änderungen vorgenommen wurden. Die in diesem Artikel enthaltenen Bilder und sonstiges Drittmaterial unterliegen ebenfalls der genannten Creative Commons Lizenz, sofern sich aus der Abbildungslegende nichts anderes ergibt. Sofern das betreffende Material nicht unter der genannten Creative Commons Lizenz steht und die betreffende Handlung nicht nach gesetzlichen Vorschriften erlaubt ist, ist für die oben aufgeführten Weiterverwendungen des Materials die Einwilligung des jeweiligen Rechteinhabers einzuholen. Weitere Details zur Lizenz entnehmen Sie bitte der Lizenzinformation auf http://creativecommons.org/licenses/by/4.0/deed.de.
License URL: https://creativecommons.org/licenses/by/4.0/

issue-copyright-statement: © Springer-Verlag GmbH Deutschland, ein Teil von Springer Nature 2021

==============================
The Coronavirus disease 2019 (COVID-19) pandemic is caused by the severe acute respiratory syndrome coronavirus 2 (SARS-CoV-2). To facilitate its own replication and avoid immune control, SARS-CoV-2 manipulates its target cells. Our results revealed that the SARS-CoV-2 non-structural protein 1 (Nsp1) plays a key role in viral immune evasion. It blocks the mRNA tunnel of the cellular ribosome, resulting in a shutdown of translation and strong attenuation of the host’s antiviral immune response.

■ SARS-CoV-2, der Verursacher der COVID-19-Pandemie, hat sich seit seinem ersten Auftreten Ende 2019 in China rasch global ausgebreitet. Bis März 2021 wurden mehr als 123 Millionen Infektionen weltweit nachgewiesen, und die Krankheit hat über 3,3 Millionen Menschen das Leben gekostet (https://coronavirus.jhu.edu/map.html, [1]). Zudem schränkt das Virus unser öffentliches Leben massiv ein und hat enorme Auswirkungen auf die Weltwirtschaft. Die sozialen und gesellschaftlichen Konsequenzen sind weitreichend und aktuell noch kaum abschätzbar.

Die COVID-19-Pandemie ist die Folge der siebten bekannten zoonotischen Übertragung von Coronaviren auf den Menschen [2]. Die ursprünglichen Wirte sind Fledermäuse, welche die Viren über unterschiedliche Zwischenwirte auf den Menschen übertragen können. Die sieben Vertreter der humanen Coronaviren gehören zu den Alpha- und Beta-Coronaviren. Beide Alpha-Coronaviren und zwei der fünf Beta-Coronaviren zirkulieren bereits lange endemisch in der menschlichen Bevölkerung und führen zu harmlosen Erkältungskrankheiten [2]. Die verbleibenden drei Vertreter der Beta-Coronaviren können dagegen schwere Atemwegserkrankungen verursachen. Bereits in den Jahren 2002 und 2003 infizierten sich etwa 8.000 Personen mit SARS-CoV-1, das eng mit SARS-CoV-2 verwandt ist. Etwa zehn Jahre später löste das besonders aggressive *middle east respiratory syndrome coronavirus* (MERS-CoV) vor allem in Ländern des nahen Ostens eine Epidemie aus. Infektionen mit diesen Coronaviren verursachen akute Atemwegserkrankungen, die bei schweren Verläufen zum Tod führen können. Dabei liegt das aktuelle SARS-CoV-2 mit einer durchschnittlichen Fallsterblichkeit von etwa ein bis drei Prozent deutlich hinter SARS-CoV-1 und MERS-CoV, die Letalitätsraten von etwa elf Prozent und 35 Prozent zeigten [2]. Jedoch wird SARS-CoV-2 effektiver übertragen als diese hochpathogenen Coronaviren und ist aber zugleich aggressiver als die endemischen Coronaviren. Die Schwere der Krankheitsverläufe und die Todesrate hängen dabei stark vom Alter der Infizierten ab. Weitere Risikofaktoren sind Übergewicht, männliches Geschlecht und Vorerkrankungen an Herz und Lunge [3]. Aktuelle Erkenntnisse deuten jedoch darauf hin, dass auch bei jüngeren Personen noch deutlich nach Ende der SARS-CoV-2-Infektion Spätfolgen auftreten können, wie beispielsweise Diabetes Typ II [4]. Derzeit ist das Ausmaß derartiger Spätfolgen noch kaum abschätzbar.

### Die menschliche Immunabwehr gegen Krankheitserreger wie SARS-CoV-2

Wir sind täglich einer Vielzahl von Viren und Bakterien ausgesetzt. Die meisten dieser Interaktionen bleiben unbemerkt, weil unser Immunsystem die Infektion verhindert oder zumindest effektiv kontrolliert. Dabei ist das „angeborene“ Immunsystem die erste, hochwirksame Verteidigungslinie gegen Krankheitserreger. Viren und andere Erreger wer-

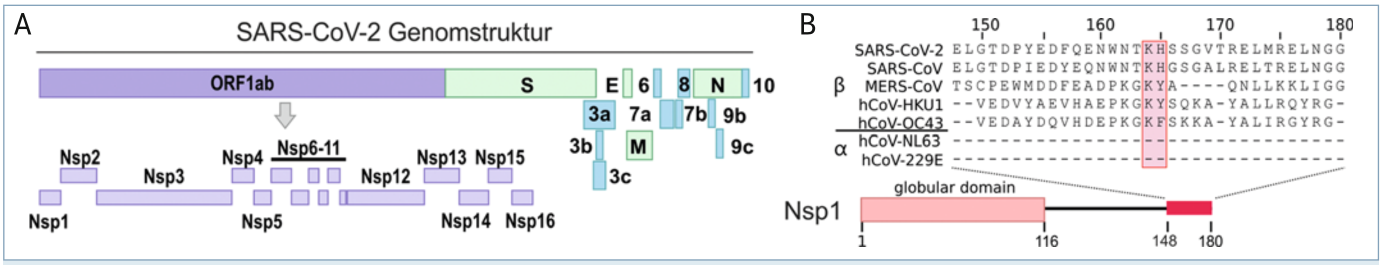

▲ **Abb. 1:** Expression und Konservierung von SARS-CoV-2-Nsp1. **A**, Schematische Darstellung des Genoms von SARS-CoV-2. Die Proteine sind durch Namen hervorgehoben [6, 7]. Lila: Polyprotein und Nichtstrukturproteine, grün: Strukturproteine, blau: akzessorische Proteine. **B**, Alignment der Aminosäuresequenz des Nsp1-C-Terminus der sieben verschiedenen humanen Coronaviren, das KH-Motiv ist farblich hervorgehoben. Darunter: Schematische Darstellung der Proteindomänen von Nsp1, der C-Terminus ist rot hervorgehoben.

Springer

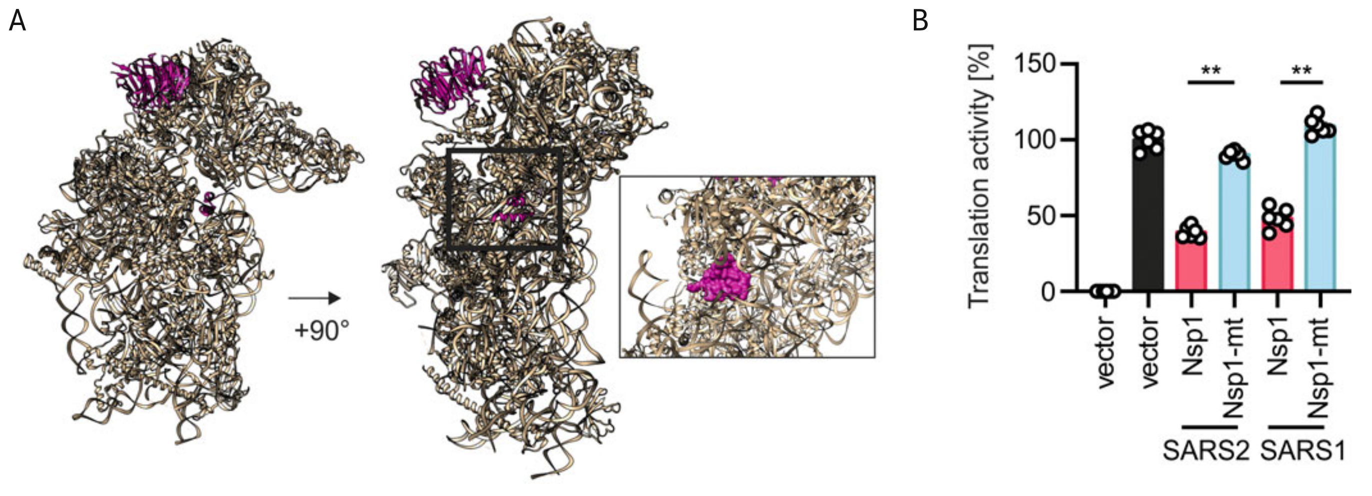

▲ **Abb. 2:** SARS-CoV-2-Nsp1 blockiert den mRNA-Eingangstunnel des Ribosoms. **A**, Strukturdarstellung von Nsp1 gebunden an die 40S-Untereinheit des menschlichen Ribosoms, dargestellt durch kryoelektronenmikroskopische Rekonstruktion im Labor von Prof. Dr. R. Beckmann [8]. PDB: 6ZLW. Lila: Nsp1, gelb: Ribosom, orange: mRNA. **B**, Nsp1 (rosa) von SARS-CoV-2 (SCoV-2) und SARS-CoV-1 (SCoV-1) inhibieren die Translation, gezeigt durch Luciferase-Reporter-mRNA-Transfektionsexperimente. 164/165AA-Mutanten von beiden Nsp1 (Nsp1-mt, blau) sind nicht mehr funktional und verhalten sich wie die Kontrolle (vector). Mittelwert ± Standardfehler, n = 6, **P < 0,001, Welch-Test. Abbildung aus [8] adaptiert.

den aufgrund ihrer spezifischen Eigenschaften (pathogenassoziierte molekulare Muster) als fremd erkannt. Die Erkennung aktiviert Signalkaskaden, die u. a. zur Ausschüttung von Botenstoffen, wie den Interferonen, führen. Diese Signalmoleküle wiederum induzieren Abwehrmechanismen, welche sowohl die infizierte Zelle als auch benachbarte Zellen in einen antiviralen Zustand versetzen [5]. Dabei kommt es zur vermehrten Produktion von Hunderten von Proteinen, von denen viele der viralen Infektion entgegenwirken. Darüber hinaus aktiviert das angeborene Immunsystem auch das „adaptive" Immunsystem, also die zweite Verteidigungslinie. Das adaptive Immunsystem „lernt" bestimmte Krankheitserreger zu erkennen und diese durch spezifische Antikörper zu neutralisieren oder mittels zytotoxischer Zellen bereits infizierte Zellen zu eliminieren. Durch das Zusammenspiel von angeborenem und adaptivem Immunsystem schafft es unser Körper, die meisten Krankheitserreger abzuwehren [5].

## SARS-CoV-2 bekämpft das angeborene Immunsystem

Viren und das Immunsystem befinden sich in einem permanenten „Wettkampf". Erfolgreiche Viren haben dabei im Laufe ihrer Evolution komplexe Strategien entwickelt, um die Erkennung durch das angeborene Immunsystem zu verhindern, die Aktivierung der Verteidigungsmechanismen zu unterdrücken oder antivirale Faktoren auszuschalten. SARS-CoV-2 ist hier besonders vielseitig [6]. Das Virus codiert etwa 30 verschiedene Proteine (**Abb. 1A**) und ein Großteil davon hilft dem Virus, die Immunabwehr zu unterdrücken und so den menschlichen Wirt erfolgreich zu infiltrieren [7]. Eine zentrale Rolle spielt dabei ein kleines Protein von 180 Aminosäuren: das Nichtstrukturprotein 1 (Nsp1). Dieses ist nicht Teil des Viruspartikels und wird in infizierten Zellen mittels Spaltung eines großen viralen Vorläuferproteins durch virale Proteasen produziert (**Abb. 1A**, [6]). Nsp1 hat zwei unterschiedliche Bereiche, eine globuläre N-terminale Domäne mit unbekannter Funktion und ein C-terminales Helix-Turn-Helix-Motiv. Dieses C-terminale Motiv findet sich bei allen Beta-Coronaviren und somit bei allen Coronaviren, die im Menschen schwere Atemwegserkrankungen verursachen, jedoch nicht bei den bislang eher harmlosen Alpha-Coronaviren (**Abb. 1B**, [8]). Wie unten beschrieben, spielt diese Domäne eine zentrale Rolle bei der Unterdrückung der antiviralen Immunantwort.

## SARS-CoV-2-Nsp1 blockiert die zellulären Proteinfabriken

Um Proteine zu produzieren, muss die Abschrift der genetischen Information, die Boten- oder *messenger* RNA (mRNA), sich an zelluläre Proteinfabriken (Ribosomen) anlagern und in Aminosäureketten übersetzt (translatiert) werden. Unsere Ergebnisse zeigen, dass Nsp1 dies verhindert [8]. Mithilfe von gefrier(kryo)elektronenmikroskopischen Aufnahmen von Nsp1-Ribosom-Komplexen konnten wir zeigen, wie Nsp1 an eine Untereinheit (40S) des Ribosoms bindet (**Abb. 2A**). Dabei besetzt das C-terminale Helix-Turn-Helix-Motiv von Nsp1 genau den Tunnel im Ribosom, der normalerweise von mRNAs zur Proteinproduktion (Translation) genutzt wird. Nsp1 verstopft wortwörtlich das Ribosom, was ein Ablesen der mRNAs und somit die *de novo*-Proteinproduktion der Zelle verhindert (**Abb. 2B**, [8]).

Eine Folge der Blockierung des Ribosoms und der Inhibition von zellulärer Translation durch Nsp1 ist, dass die Zelle keine Proteine mehr produzieren kann. Insbesondere sind davon auch die Faktoren betroffen, die notwendig für die angeborene Immunabwehr sind. Wichtige Abwehrmechanismen beruhen darauf, dass nach Erkennung des Virus Alarmbotenstoffe, wie z. B. verschiedene Interferone oder Interleukine, synthetisiert werden. Unsere Ergebnisse zeigen, dass Nsp1 die Produktion dieser Botenstoffe effektiv unterdrückt. Zwar wird der Erreger noch erkannt und die mRNA, welche die genetische Information für diese Botenstoffe trägt, vermehrt gebildet (**Abb. 3**). Allerdings kann die Zelle nicht mehr darauf reagieren, weil diese mRNAs nicht mehr vom Ribosom abgelesen und in Aminosäureketten übersetzt werden können. Nsp1 verhindert somit durch Inhibition der Translation die Produktion wichtiger Alarmsignale des angeborenen Immunsystems [8]. Dadurch kommt es nicht zur Ausschüttung von antiviralen Botenstoffen und auch das adaptive Immunsystem wird schlechter aktiviert.

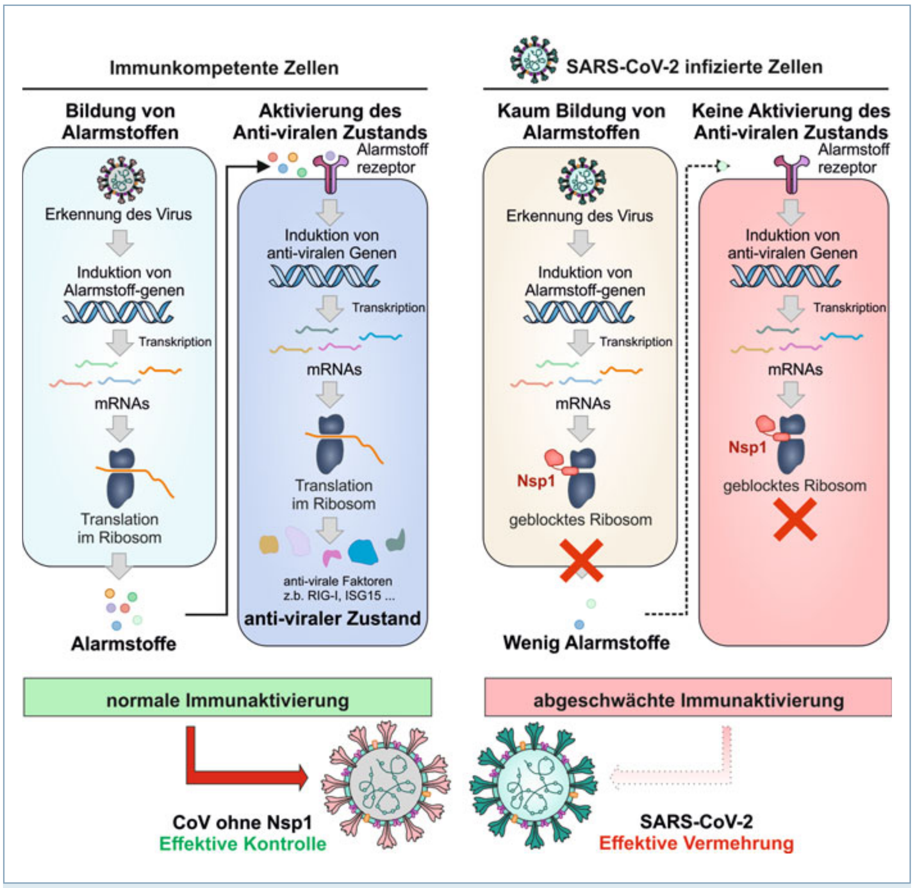

▲ **Abb. 3:** Schematische Darstellung der Funktion von Nsp1. In einer gesunden Zelle (blauer Hintergrund) können mRNAs, welche die genetische Information für Alarmstoffe und antivirale Faktoren enthalten, durch das Ribosom in Proteine übersetzt werden. Dies ermöglicht die Etablierung eines antiviralen Zustands und eine normale Immunantwort gegen eindringende Krankheitserreger. In einer mit SARS-CoV-2 infizierten Zelle (roter Hintergrund) blockiert Nsp1 das Ribosom. Somit können Alarmstoffe und antivirale Faktoren nicht mehr translatiert und kein antiviraler Zustand hergestellt werden. Die Immunantwort gegen das Virus ist abgeschwächt.

Aber damit nicht genug: Nsp1 unterdrückt nicht nur in die Produktion der Alarmstoffe, sondern verringert auch deren Effekte, nämlich die Produktion von Proteinen mit direkter antiviraler Aktivität, den Effektoren. Viele Effektoren werden in Gegenwart von Interferonen vermehrt gebildet und deswegen auch als interferonstimulierte Gene (ISGs) bezeichnet. Wir haben einige davon untersucht und konnten zeigen, dass Nsp1 z. B. die Synthese von RIG-I (*retinoic acid inducible gene I*), das bei der Erkennung von mehreren RNA-Viren eine zentrale Rolle spielt, sowie von ISG15 (*interferon-stimulated gene 15*), das breite antivirale Aktivität hat, hemmt (**Abb. 3**). Somit führt Nsp1 zum einen dazu, dass SARS-CoV-2 infizierte Zellen mehr infektiöse Viruspartikel produzieren und zum anderen, dass noch uninfizierte benachbarte Zellen empfänglich gegenüber der Infektion bleiben. Das Virus kann sich somit besser vermehren und ausbreiten.

Alle beschriebenen Effekte sind abhängig von der Fähigkeit von Nsp1, die Translation der mRNAs durch das Ribosom zu blockieren. Eine Nsp1-Mutante, bei der lediglich zwei Aminosäuren verändert wurden (164/165AA, Nsp1-mt), war nicht mehr in der Lage, an das Ribosom zu binden und die Immunantwort zu unterdrücken.

Insgesamt verhindert Nsp1 somit, dass infizierte Zellen auf die Virusinfektion reagieren können [8]. Die Immunantwort wird abgeschwächt und der Erreger kann sich effektiv ausbreiten.

## Bedeutung und offene Fragen

Die Blockierung des zellulären Ribosoms und die damit verbundene Unterdrückung der Proteinproduktion führen dazu, dass antivirale Signalmoleküle und Effektoren auch nach Detektion des Virus durch das Immunsystem nicht mehr gebildet werden können. Dies ist ein wichtiger Mechanismus, durch den sich SARS-CoV-2 der Kontrolle durch das Immunsystem entzieht – aber bei weitem nicht der einzige. Aktuelle Untersuchungen zeigen, dass viele der etwa 30 Proteine von SARS-CoV-2 Immunfunktionen hemmen [7]. Die Aufklärung der relativen Bedeutung der verschiedenen Mechanismen für die Vermehrung von SARS-CoV-2 und das Krankheitsbild von COVID-19 in Menschen erfordert weitere Untersuchungen.

Unser Ergebnis, dass Nsp1 die Proteinproduktion in SARS-CoV-2-infizierten Zellen verhindert, wirft auch eine Reihe von Fragen auf. Viren sind selbst auf das zelluläre Ribosom angewiesen, um ihre eigenen Proteine zu bilden und müssen zahlreiche zelluläre Proteine benutzen, um sich zu vermehren. Wie es SARS-CoV-2 schafft, dass die eigenen mRNAs auch in Gegenwart von Nsp1 in virale Proteine abgelesen werden können, ist noch nicht abschließend geklärt. Weiterhin ist unklar, ob Nsp1 die Translation aller zellulärer Proteine verhindert oder manche mRNAs effizienter hemmt als andere. Die Blockierung der zellulären Translation verhindert nicht nur die Immunantwort, sondern hat auch zahlreiche andere Effekte. Letztendlich hängt auch das Überleben der infizierten Zelle von einer funktionalen Proteinproduktion ab. Daher wäre es interessant herauszufinden, ob die Blockade der Ribosomen durch Nsp1 zum Tod von Lungenzellen in COVID-19-Patienten beiträgt. Interessanterweise konnten auch einige Varianten von Nsp1 in SARS-CoV-2-infizierten Personen nachgewiesen werden. Aktuell erforschen wir daher, welchen Einfluss diese Varianten auf die Funktion von Nsp1 und den klinischen Verlauf der Infektion haben.

Unsere Studie liefert einige neue Ansatzpunkte zur Bekämpfung von SARS-CoV-2. Eine interessante Frage ist, ob SARS-CoV-2-Varianten, die ein mutiertes Nsp1-Protein besitzen (beispielsweise 164/165AA), welches nicht mehr ans Ribosom bindet, effektiv durch das Immunsystem kontrolliert werden. Derartige abgeschwächte Varianten könnten potenziell sogar die Basis für rationale Lebendimpfstoffe bilden. Unsere Kenntnisse der molekularen Struktur von Nsp1 am Ribosom könnten neue Therapiemöglichkeiten gegen SARS-CoV-2 eröffnen. Beispielsweise sollten kleine Moleküle, Peptide oder RNAs, die spezifisch die Interaktion zwischen Nsp1 und dem Ribosom verhindern, die Blockade des angeborenen Immunsystems aufheben. Somit kann das Virus besser durch das

Immunsystem kontrolliert und die Ausbreitung verhindert werden.

## AUTOREN

Foto: Elvira Eberhardt

### Frank Kirchhoff

Foto: Elvira Eberhardt

### Konstantin Sparrer

Hier steht
eine Anzeige.

Springer


Funding note: Open Access funding enabled and organized by Projekt DEAL.

Danksagungen

Die Autoren danken allen an der Studie beteiligten Wissenschaftlern, insbesondere Roland Beckmann und seiner Gruppe (LMU München).

Frank Kirchhoff Biologiestudium an der Universität Göttingen. 1991 Promotion. 1991–1994 Postdoc am New England Primate Research Center, Harvard Medical School, Boston, USA. 1994–2001 Arbeitsgruppenleiter am Lehrstuhl für Virologie, Universität Erlangen-Nürnberg. 2001–2009 Professor und Arbeitsgruppenleiter am Institut für Virologie und seit 2009 Direktor des Instituts für Molekulare Virologie am Universitätsklinikum Ulm.

Konstantin Sparrer 2005–2010 Studium der Chemie und Biochemie an der LMU München. 2013 Promotion. 2013–2014 Postdoktorand am Max von Pettenkofer-Institut, München. 2015–2017 Postdoktorand an der Harvard Medical School, Boston, MA, und University of Chicago, IL, USA. Seit 2018 Juniorgruppenleiter am Institut für Molekulare Virologie am Universitätsklinikum Ulm.


Literatur

[1] Dong E Du H Gardner L An interactive web-based dashboard to track COVID-19 in real time Lancet Infect Dis 2020 20 533 534 10.1016/S1473-3099(20)30120-1 32087114 PMC7159018
[2] Andersen KG Rambaut A Lipkin WI The proximal origin of SARS-CoV-2 Nat Med 2020 26 450 452 10.1038/s41591-020-0820-9 32284615 PMC7095063
[3] CDC (2020) Evidence used to update the list of underlying medical conditions that increase a person’s risk of severe illness from COVID-19. https://www.cdc.gov/coronavirus/2019-ncov/need-extra-precautions/evidence-table.html 34009770
[4] Müller JA Groß R Conzelmann C SARS-CoV-2 infects and replicates in cells of the human endocrine and exocrine pancreas Nat Metab 2021 3 149 165 10.1038/s42255-021-00347-1 33536639
[5] Sparrer KMJ Gack MU Intracellular detection of viral nucleic acids Curr Opin Microbiol 2015 26 1 9 10.1016/j.mib.2015.03.001 25795286 PMC5084527
[6] V’kovski P Kratzel A Steiner S Coronavirus biology and replication: implications for SARS-CoV-2 Nat Rev Microbiol 2020 19 155 170 10.1038/s41579-020-00468-6 33116300 PMC7592455
[7] Hayn M, Hirschenberger M, Koepke L et al. (2020) Systematic analysis of innate immune antagonism reveals vulnerabilities of SARS-CoV-2. Cell Rep 10912610.1016/j.celrep.2021.109126 PMC8078906 33974846
[8] Thoms M Buschauer R Ameismeier M Structural basis for translational shutdown and immune evasion by the Nsp1 protein of SARS-CoV-2 Science 2020 369 eabc8665 10.1126/science.abc8665 PMC7402621 32680882
